# Comparison of Dentin Bond Strength Between a Bioactive Resin-Modified Glass Ionomer Cement and a Conventional Self-Adhesive Resin Cement: An In Vitro Study

**DOI:** 10.7759/cureus.115811

**Published:** 2026-09-05

**Authors:** Ali Y Yousef, Mirza M Allaf, Mohammad Y Hajeer

**Affiliations:** 1 Department of Fixed Prosthodontics, Faculty of Dentistry, University of Damascus, Damascus, SYR; 2 Department of Orthodontics, Faculty of Dentistry, University of Damascus, Damascus, SYR

**Keywords:** bioactive resin-modified glass ionomer cement, bioactivity, bond strength, conventional self-adhesive resin cement, self-adhesive cement, shear bond strength

## Abstract

Background and aim: Bond strength is one of the most important requirements for luting cements. Recently, bioactivity has been added to some cements to improve bond strength and mechanical and biological properties. This study aimed to compare the dentin shear bond strength between a bioactive resin-modified glass ionomer cement and a conventional self-adhesive resin cement. The null hypothesis was that there was no significant difference in dentin shear bond strength between the bioactive resin-modified glass ionomer cement and the conventional self-adhesive resin cement.

Materials and methods: A total of 24 freshly extracted and collected mandibular premolars were embedded in acrylic bases, and the occlusal surface was shortened by 2 mm to expose the dentin. The specimens were divided equally into the following two groups: group A, the bioactive resin-modified glass ionomer cement (RMGIC) group (ACTIVA; Watertown, MA: Pulpdent), and group B, the conventional self-adhesive resin cement (Breeze; Wallingford, CT: Pentron). A total of 24 discs of the two cements were bonded to the occlusal dentin surface. The specimens were immersed in artificial saliva for 30 days and then subjected to 1000 thermal cycles at temperatures of 5°-55°C. The shear bond strength (SBS) test was then performed. The independent-samples t-test was used.

Results: The mean shear bond strength (SBS) of ACTIVA cement (group A) was 7.65±1.11 MPa, which was significantly higher than the mean of the Breeze group (group B) at 5.23±0.82 MPa (t=6.065, p<0.001), showing a mean difference of 2.42 MPa. Data in both groups were normally distributed, as indicated by the Shapiro-Wilk test (p>0.05). Consequently, the null hypothesis of no significant difference in dentin shear bond strength between the two materials was rejected.

Conclusions: The dentin bond strength of bioactive resin-modified glass ionomer cement (RMGIC) was significantly higher than that of conventional self-adhesive resin cement. This finding underscores the effectiveness of bioactive glass components in stimulating hydroxyapatite formation, thereby strengthening chemical adhesion to dentin. Bioactive RMGIC is therefore recommended for luting restorations on vital abutments with compromised mechanical retention or large areas of exposed dentin, such as short or over-tapered preparations.

## Introduction

Dental cements must meet several requirements, including strong adhesion to dental tissues and restorative materials, high mechanical properties, resistance to dissolution, good film thickness, biocompatibility, good working and setting time, and aesthetic properties. Resin cements have superior mechanical and esthetic properties compared with other acid-base cements, and their solubility is almost zero [1]. However, they cause postoperative sensitivity because of acid etching and the permeability of free unpolymerized monomers [2-4]; they are also sensitive and a difficult technique that requires several steps before adhesion [5].

Self-adhesive resin cements were developed to overcome these disadvantages. These cements contain the etchant, primer, adhesive, and cement in the same package, and they combine the high mechanical properties of resin cements with superior biological properties [6]. Recently, bioactive cements have been developed to improve the mechanical and biological properties of luting cements, such as glass ionomer cement (GIC), resin-modified glass ionomer cement (RMGIC), and resin cement [7,8].

Bioactivity is the ability of a material like calcium silicate or calcium aluminate to form hydroxyapatite on its surface when it contacts phosphate ions, and this differs from biointeractivity, which is the ability of a material to release calcium and fluoride ions that enter saliva and cause remineralization of adjacent teeth [9,10].

ACTIVA cement is a bioactive resin-modified glass ionomer cement, developed in 2013. It is a triple-cure, self-adhesive cement composed of a flexible, shock-absorbing ionic resin matrix (polybutadiene-modified urethane dimethacrylate) and bioactive glass fillers [11,12]. According to the manufacturer, this cement has physical and chemical properties similar to natural teeth. It has the ability to form hydroxyapatite and bond the resin to the tooth. The setting of this cement is done through the following three mechanisms: acid-base reaction, chemical curing, and photocuring [13].

Formation of hydroxyapatite and increased chemical adhesion to dentin are beneficial in situations where the role of the cement is particularly important in resisting axial and oblique forces due to poor mechanical retention, such as short abutments, over-prepared taper abutments, and non-retentive partial preparations [5,14,15].

## Materials and methods

**Figure 1:**
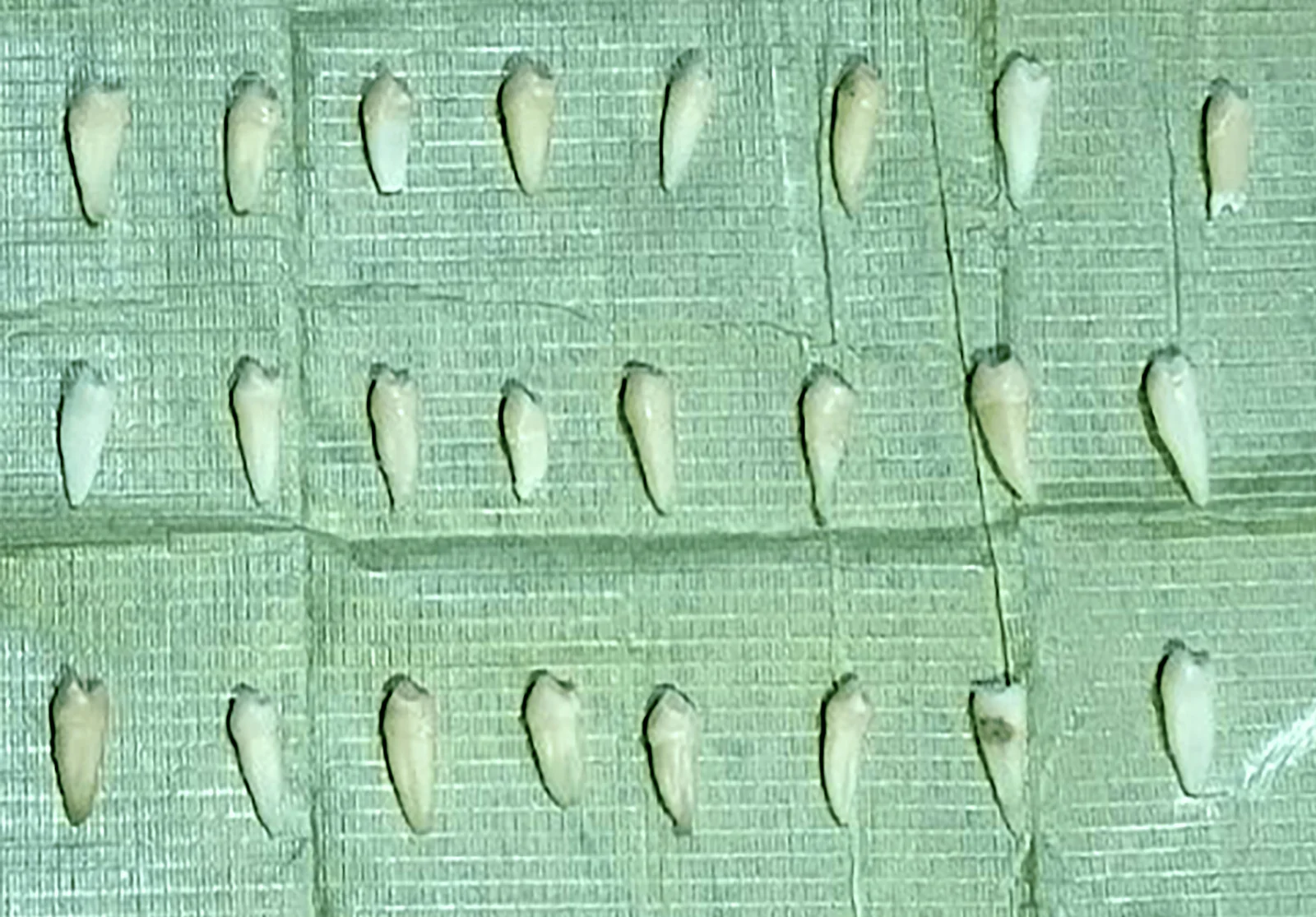
The sample of extracted teeth.

A total of 24 freshly extracted, decay-free, and unfilled mandibular premolars were collected for this study (Figure 1). The use of extracted premolars for this part of the research work was approved by the Biomedical Research Ethics Committee of the University of Damascus (#DN-230426-719). The mandibular premolars were extracted for clear clinical indications, such as orthodontic treatment or advanced periodontal disease. Before extraction, patients provided explicit informed consent for the use of their teeth in scientific research. Patient anonymity was strictly maintained, rendering all specimens completely untraceable. The premolars were stored in artificial saliva for 24 h and then anchored in acrylic bases, with the acrylic covering the root up to 2 mm before the cementoenamel junction.

**Figure 2:**
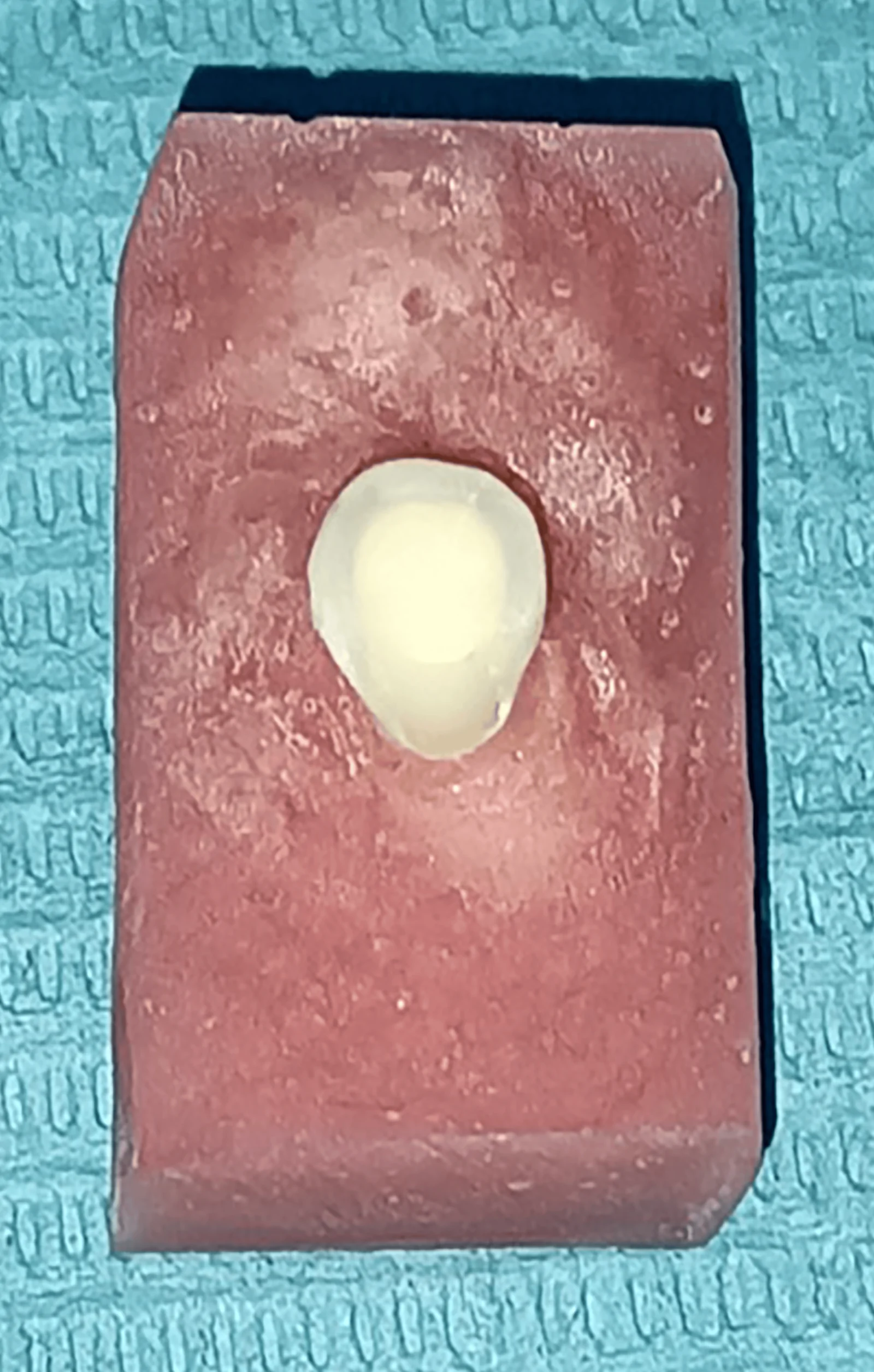
The flat occlusal surface and the exposed dentin.

The occlusal surface was shortened by 2 mm to expose the dentin tissue. A sectioning disc was used for 1 min to make the occlusal surface flat, which was then smoothed and polished with abrasive paper for 30 s (Figure 2). The teeth were then washed, dried, and divided equally into two groups.

**Figure 3:**
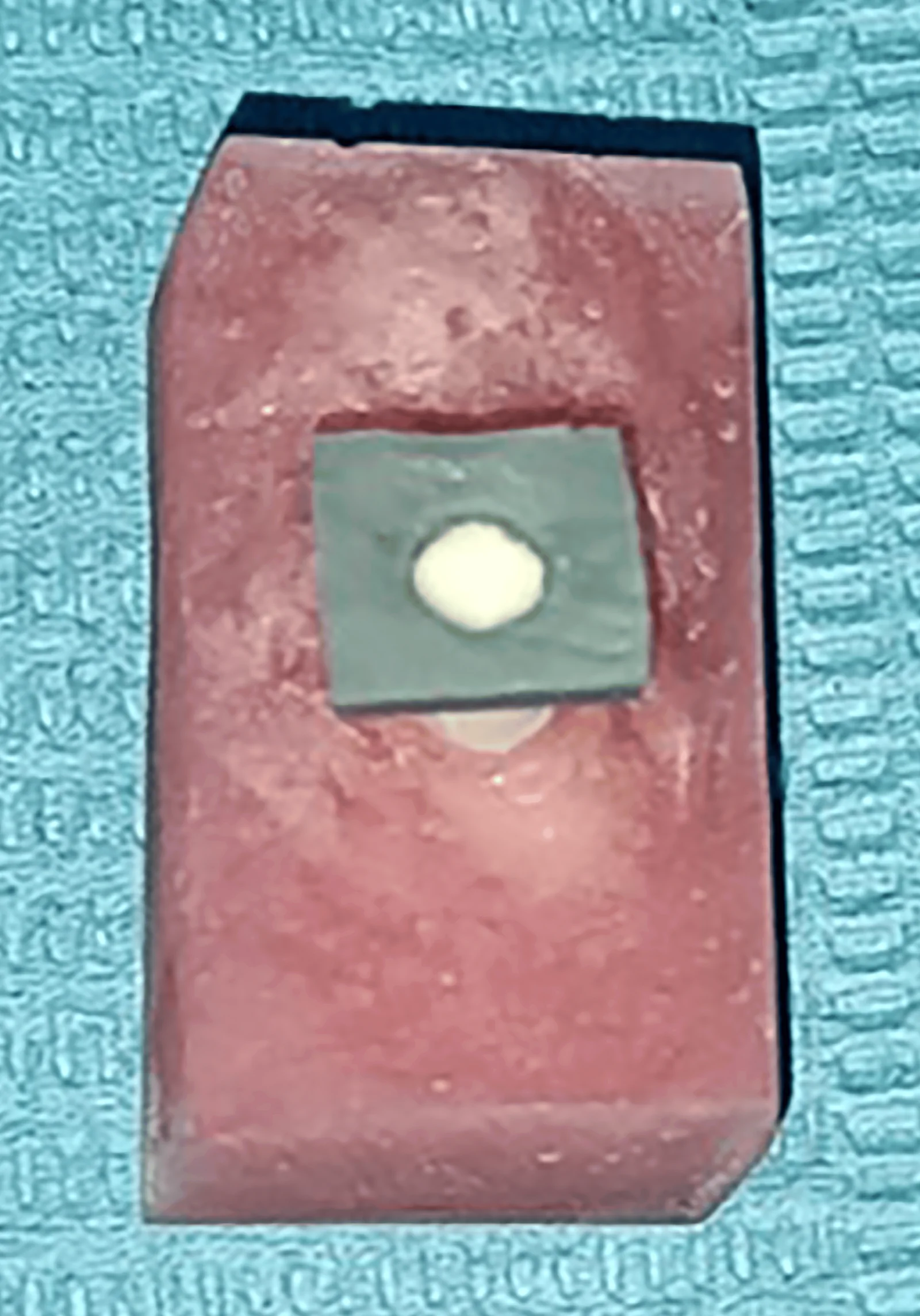
Silicone mold on the occlusal surface of the premolar, with the empty circle placed over the exposed dentin.

A disk (4 mm diameter × 2 mm thickness) was designed by computer-aided design/computer-aided manufacturing (CAD/CAM) and fabricated from polymethyl methacrylate (PMMA). This disk was then used to form a mold from condensation silicone (Zetaplus; Marl, Germany: Zhermack). The silicone mold was placed on the occlusal surface of the teeth, and the cement was injected into it, according to the allocated group (n=12) as follows: group A - bioactive RMGIC (ACTIVA; Watertown, MA: Pulpdent); group B - conventional self-adhesive resin cement (Breeze; Wallingford, CT: Pentron) (Figure 3).

**Figure 4:**
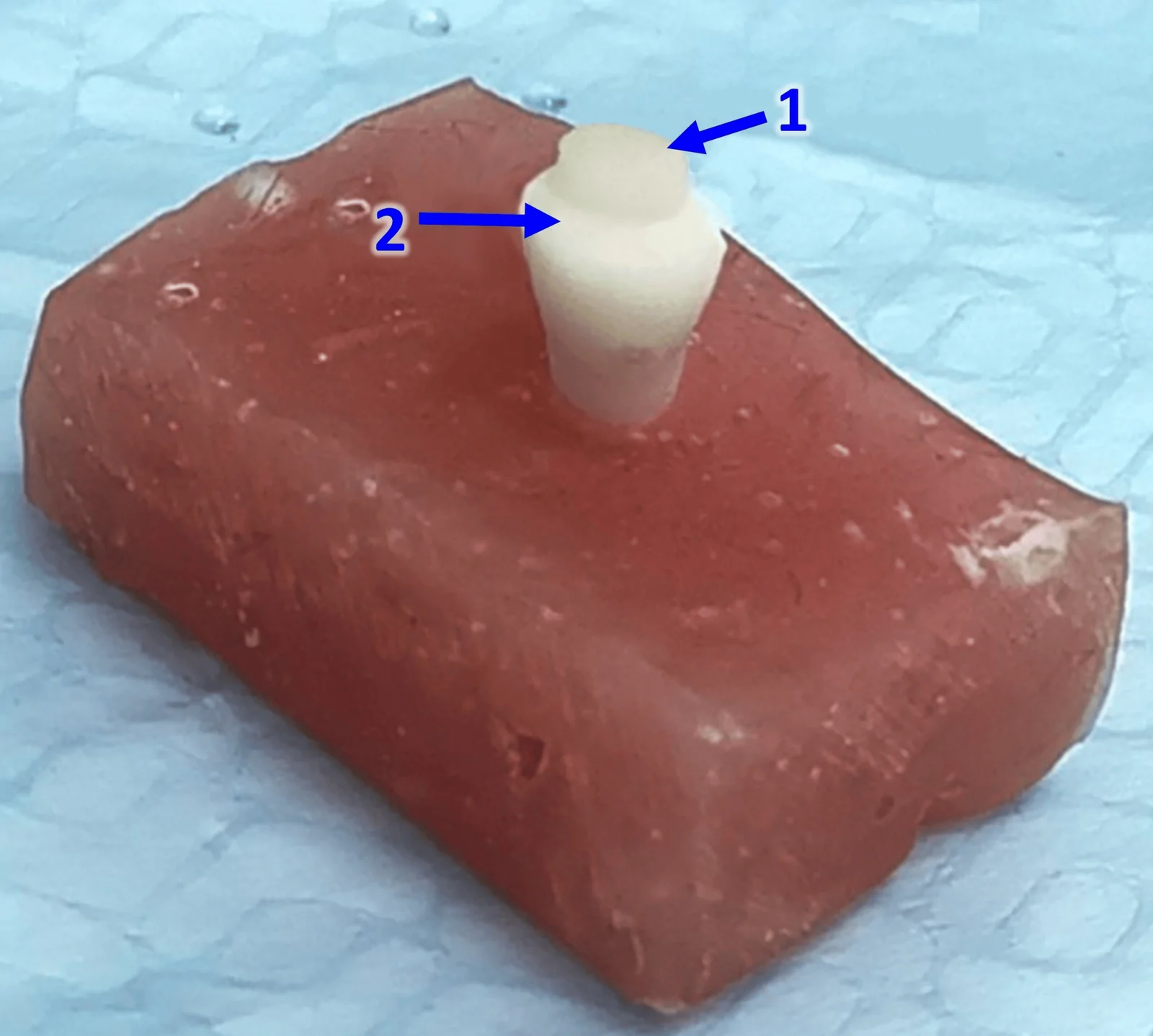
Specimen of cement disc bonded to the dentin surface. 1: cement disc; 2: dentin surface

A small glass plate was placed over the occlusal surface of the mold, and primary light-curing was done for 3 s. Excess material was removed using a small brush, followed by final light-curing for 40 s (Figure 4). The samples were then left for 5 min undisturbed to ensure complete polymerization before removing the mold.

**Figure 5:**
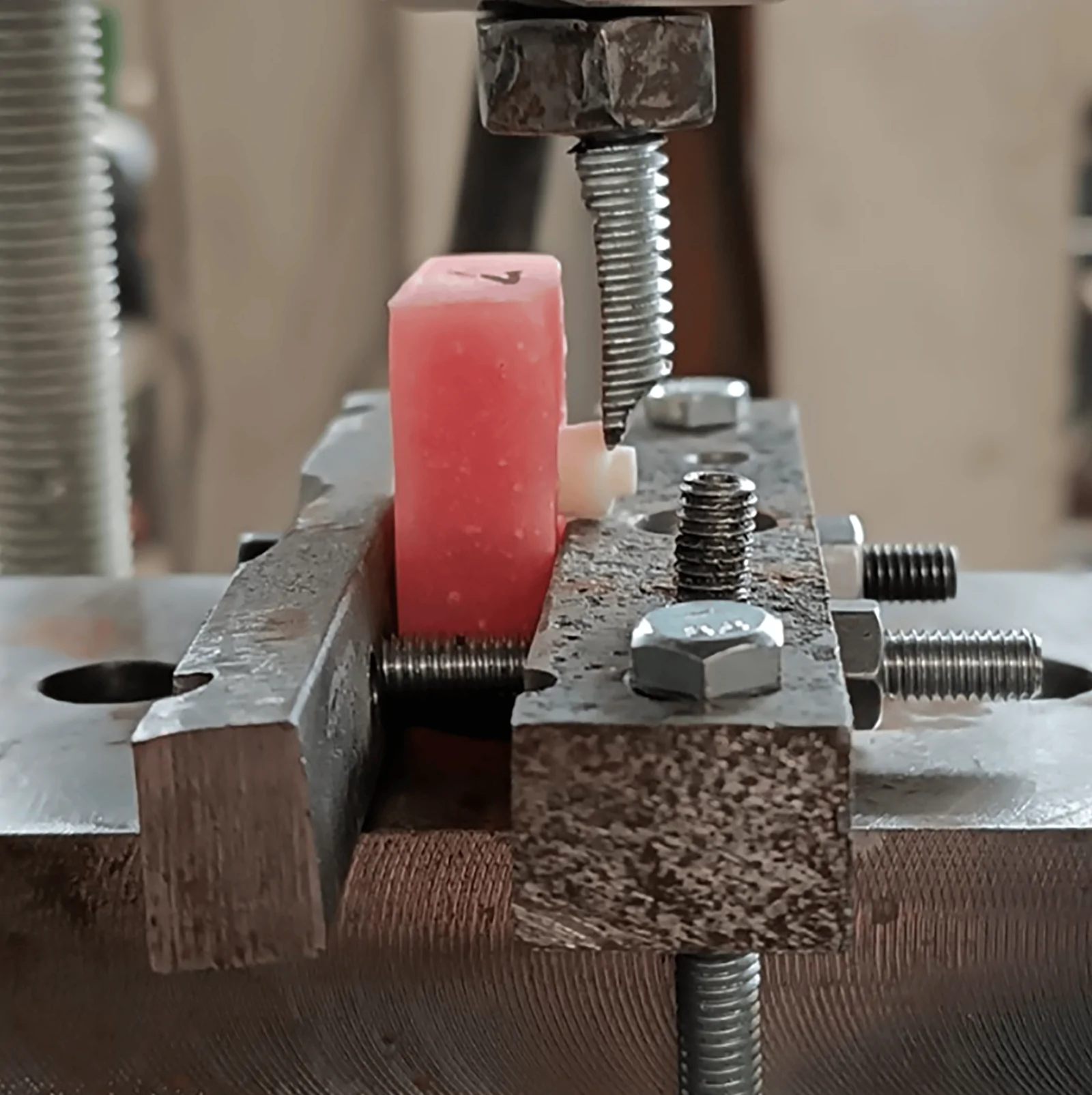
Shear bond strength (SBS) testing setting.

The samples were stored in artificial saliva (NeutraSal; Bridgewater, NJ: OraPharma, Inc.), which contains phosphate ions, for 30 days and then subjected to 1000 thermocycles at temperatures of 5°-55°C. The shear bond strength (SBS) test was performed using a universal testing machine (Faculty of Mechanical and Electrical Engineering, Damascus University) at a speed of 1 mm/min (Figure 5).

Statistical analysis

SPSS version 26 (Armonk, NY: IBM Corp) was used to analyze the data in the current study. The Shapiro-Wilk test was used to assess the normal distribution of the data, and the results showed that shear bond strength was normally distributed. The statistical results were calculated at a significance level of 0.05, and an independent-samples t-test was used to compare shear bond strength forces between group A and group B.

## Results

The Shapiro-Wilk test was used to assess the normality of the data distribution of shear bond strength for each of the two groups (Table 1). Table 1 shows that the p-value was greater than 0.05, suggesting that the shear bond strength data in the study samples followed a normal distribution. Therefore, a parametric statistical test was applied.

**Table 1:** Assessment of normality of the shear bond strength variables. Shapiro-Wilk test was used for statistical analysis.

Group A		Group B	
Test result	p-Value	Test result	p-Value
0.884	0.099	0.925	0.334

The mean shear bond strength was 7.65±1.11 MPa and 5.23±0.82 MPa for groups A and B, respectively. An independent-samples t-test was used to compare shear bond strength between the two study groups (Table 2). The results of the independent-samples t-test showed a statistically significant difference between the two groups (p<0.001), with the difference being in favor of group A.

**Table 2:** Descriptive statistics for the shear bond strength in the two groups along with the significance of statistical testing.

Comparison between the two groups	n	Mean (MPa)	Standard deviation (SD)	Mean difference	T-value	p-Value
Group A	12	7.65	1.11	2.42	6.065	<0.001
Group B	12	5.23	0.82			

## Discussion

RMGIC was introduced to combine the advantages of glass ionomer cement (GIC) with those of resin cement, resulting in lower solubility and higher mechanical properties than GIC, while retaining the biological properties and fluoride release of GIC [1]. However, it has many disadvantages, such as water absorption and swelling, which may cause cracks or fractures inside the ceramic [1]; its bond strength and mechanical properties are still lower than resin cements, and its solubility is higher [16,17].

By developing bioactive properties, bioactive RMGIC possesses lower water absorption and solubility and better fluoride release, with improved mechanical properties [7,13]. Bioactivity also stimulates the formation of hydroxyapatite on the dentin surface adjacent to the cement, increasing the strength of the chemical bond [18].

Therefore, this study compared ACTIVA cement with traditional self-adhesive cement, which is an indicated option for adhesion in cases with compromised retention, such as high-taper walls and partial preparations, especially those with high dentin exposure [5], as adhesion between dentin and traditional resin cements is difficult and complex due to the organic content of dentin and the presence of a smear layer [19].

A shear bond strength test was performed to assess bond strength because it is the most commonly used and practical test in studies, making specimen preparation more accurate [20,21]. The sample size was also chosen to be larger than in previous in vitro studies that investigated the shear bond strength of resin cements [21,22].

The specimens were smoothed using the same speed and methodology to standardize surface roughness as well as the dimensions of the discs, because surface area and roughness are factors that affect retention and adhesion [23,24]. The specimens were immersed in artificial saliva for 30 days to come into contact with phosphate ions, which is a major factor in stimulating hydroxyapatite formation in ACTIVA cement [25], and 1000 thermal cycles were performed to match one month of oral aging [26,27]. This number has been adopted in several in vitro studies investigating the shear bond strength of cements [28,29].

The mean SBS of ACTIVA cement was 7.65±1.11 MPa, which was significantly higher than the mean of the Breeze group at 5.23±0.82 MPa. The superior SBS of the bioactive cement to dentin may be attributed to its bioactivity, hydroxyapatite formation, and increased chemical bonding. These results differed from both the study by Alkhudhairy et al. [8], which concluded that the SBS of ACTIVA was lower when compared to conventional dual-cure resin cements Calibra and Variolink II on dentin surfaces treated with the erbium, chromium-doped yttrium, scandium, gallium, and garnet (Er,Cr:YSGG) laser (ECL), and the study by Vohra et al., who compared the SBS of ACTIVA and the conventional resin cement Nexus 3, and reported that the values ​​were similar between ACTIVA and Nexus 3 [30].

This difference from other studies may be attributed to a different adhesion pattern, where total etch and an adhesive were used with the other cements, unlike the current study, in which the resin cements were self-adhesive, or to a different research methodology, in which the specimens in this study were immersed in artificial saliva for a month to stimulate biological activity, unlike the other studies.

Limitations

This study was performed in vitro. Although the odontoblast remains active for 30 days after extraction and has the ability for hydroxyapatite formation, its effectiveness may differ from the oral environment, which may affect the bond strength between the teeth [25]. An additional limitation is that the selected teeth did not completely satisfy the study objectives.

## Conclusions

Within the limitations of this study, the dentin bond strength of bioactive resin-modified glass ionomer cement (RMGIC) was higher than that of conventional self-adhesive resin cement, and that often emphasizes the effectiveness of the added bioactive components and their ability to bond chemically to dentin and increase the bond strength of RMGICs and make them superior to the conventional ones, in addition to their favorable biological properties, so it is recommended to use bioactive RMGIC with adhesion on vital abutments where the dentin exposure area is large and the retention is compromised, such as short abutments, over-prepared taper abutments, or long-span bridges.
